# WHO steps up its role in health emergencies

**DOI:** 10.2471/BLT.15.031215

**Published:** 2015-12-01

## Abstract

The World Health Organization is on the brink of a major transformation into an agency that is fully mandated and equipped to respond to outbreaks and humanitarian emergencies. David Nabarro talks to Fiona Fleck.

Q: How did you first become interested in working in emergencies?

A: When I started out as a physician during the early 1970s, I wanted to work with communities, rather than in hospitals, particularly in places where people were unable to access health services. I worked in Kurdistan in northern Iraq in 1974, in rural Nepal, eastern India and then Bangladesh. But my interest in community health was there before I studied medicine. I wanted to understand what was needed for people to be less distressed, and not to suffer when ill – wherever they lived, and whether they were poor or wealthy. When I worked in areas affected by conflict I realized that the challenges are the same as anywhere else but that it’s more difficult to ensure that people can access the health care they need. 

Q: Can you tell us about your appointment as the UN Secretary-General’s special envoy for Ebola in 2014 and how the outbreak triggered soul-searching and reconsideration of the way health emergencies are addressed? 

A: The outbreak was bigger than anything we’d seen before and moving so fast that organizations had to develop and revise their action plans while they were being implemented. The presidents of the most affected countries were asking the UN to play a leading role in ensuring they were properly supported. They felt abandoned. Flights to their countries had been cut. They weren’t receiving the help they needed. I was working with [WHO Director-General] Margaret Chan, the Secretary-General [Ban Ki-moon] and senior advisers as they sought a massive scale-up in support of the affected countries. The Secretary-General proposed a totally new mechanism, based on what is used for UN peacekeeping operations, to give extra muscle and coordination to the international response. It became known as the UN Mission for Ebola Emergency Response or UNMEER for short. UNMEER was approved by the UN General Assembly and endorsed by the UN Security Council in the middle of September 2014: it was implemented in record time. It was developed at a time when we did not know how big the outbreak would become, with some projections that more than a million people would become infected. 

Q: The Report of the Ebola Interim Assessment Panel released this year found that coordination was weak between WHO and its UN and nongovernmental partners on many levels – financial, logistical etc. – during the outbreak and that WHO lacked staff capacity to respond adequately. How can the response to such emergencies be more effective in future?

A: There were concerns about whether WHO sounded the alarm soon enough in the early months of 2014 and others are examining this. Since August 2014 WHO has consistently provided the technical guidance, analytical expertise and capacity to lead and this has been valued by all involved in the response. At the same time, the UN helped to bring together different UN agencies and partners, each with their sets of skills and can do this in future. As a new entity offering additional capacity for overall leadership, liaison with governments, donor engagement and logistical support, UNMEER was unprecedented and unlike any other UN operation in which I have been involved.

“It is never one organization alone that responds to an emergency, partnerships are essential.”

Q: In what sense? 

A: If you get it right, a leadership and coordination body such as UNMEER can bring the players together like the conductor of an orchestra. Individual musicians may play beautiful melodies, but the power of harmony emerges through the skills of the conductor. We need to do more of this at the UN. There are thousands of nongovernmental groups – and some businesses as well – that yearn for effective coordination and clear direction. It is never one organization alone that responds to an emergency, partnerships are essential. 

Q: One of the challenges for a more effective response to outbreaks and health emergencies cited in the interim panel assessment was WHO’s decentralized governance structure allowing regional and country offices a measure of autonomy. How does the new approach address this potential bottleneck for responding to health emergencies?

A: The advisory group proposes a new WHO programme on outbreaks and emergencies that spans the whole organization and answers to one point of authority, the Director-General. The new programme should have a single platform that works across the organization and supports operations – i.e. getting people and materials quickly and safely to where they are needed, supporting them while they do their work and enabling them to travel out when necessary. Sometimes WHO staff have been ready to deploy to where they are needed but have been delayed because there was no capacity across the Organization to mobilize them. 

Q: How can WHO achieve a more effective response?

A: The unified programme and platform should use procedures that are specially designed for outbreak and emergency work. This means creating a culture of rapid response that is new for much of WHO, but which is routinely practised by humanitarian organizations. They have well-developed inter-relations, they know what to expect of each other, and engage on the basis of shared values, trust and mutual respect. Typically WHO’s way of working is to provide advice. The new culture means taking responsibility for ensuring that essential actions – leadership, coordination, information systems, communication – are undertaken. It also means WHO acting always as a neutral and independent organization, giving first priority to people whose lives and health are at risk. When a potential outbreak or emergency is picked up, WHO must be counted on to assess the risks, raise the alarm, and respond robustly and quickly – all the time drawing on capacities of partner organizations. The unified programme and platform will need its own business processes too, so as to be able to engage personnel, establish services and move supplies and finance quickly and precisely. Building these partnerships takes time because trust cannot be built overnight and is best done when people are not stretched during an emergency. It would be great if in a few years’ time WHO is regarded as an equal by the major organizations that respond to health risks in humanitarian settings. The proposals are in the advisory group’s first report to the Director-General and she will now be exploring ways in which they can be implemented. Making the shift will not be easy, but it has to happen. This is a change that WHO’s Member States and its partners want.

Q: WHO is an established player in responding to outbreaks. How will the new programme change the Organization’s approach and that of its UN partners to responding to natural disasters and conflicts? 

 A: The new approach will provide WHO with the tools and resources to respond more independently and proactively to emergencies than it has been able to do so far. Ideally, WHO should be able to use its sentinel (surveillance) network to determine independently whether a health outbreak constitutes an outbreak of international concern and then mobilize a response, together with partner organizations, at the appropriate level – which could be a country-level response for smaller outbreaks or a regional-level or international response for larger ones. 

Q: Will the new approach lead to more prevention efforts? 

 A: Preparedness is key to addressing outbreaks early on. By investing in better basic health systems and surveillance networks, we should be able to detect health outbreaks much earlier. Better prevention is essential as well. If your hospital is not built in an earthquake-resistant manner, it will be reduced to rubble when an earthquake hits and you will lose your health infrastructure the moment you need it most.

“The new approach represents a radical shift and is seen to be urgent and necessary by leaders the world over.”

Q: Ten years ago you led a WHO programme called Health Action in Crisis responding to health emergencies, while others were building an outbreak response capacity at WHO. Those initiatives were wound down. Are they being revived now in the new programme?

A: I left WHO in 2005. I understand that the WHO budget became very tight between 2006 and 2009. This led to cut-backs. Our advisory group on outbreaks and emergencies proposes a new programme that enables WHO to fulfil expectations and to do so in a predictable and dependable way. That means harnessing the existing capacity to respond to health emergencies within the Organization and delivering well. This change is urgently needed because the Organization is too easily stretched when responding to outbreaks and emergencies. As a result, it is neither predictable nor dependable in these circumstances, and that means that communities and the world are vulnerable. The new approach represents a radical shift and is seen to be urgent and necessary by leaders the world over. There is no going back now. 

Q: In addition to the US$ 100 million contingency fund and pandemic emergency financing facility, how will the new programme be funded?

A: Our advisory group is proposing a strategy for mobilizing resources. We have a number of ideas that are still under discussion. No other international health organization can work in major outbreaks of disease or in health emergencies, especially cross-border situations, apart from WHO. Sometimes people say “WHO is the first line of defence against global health emergencies, and it’s the last defence” meaning that if WHO is not strong and able to deal with these issues, the world is vulnerable. WHO needs to be able to do this work well. But WHO cannot meet the world’s expectations of it without the necessary budget. That’s why it has been extremely difficult for those running the Organization to respond to needs. The money tends to comes in when there is a crisis. But you need to do the work between crises because that’s when you build capacity. Now is the moment when it will be possible for the Organization to be given the resources and support it needs to function adequately in outbreaks and emergencies.

